# Genetic separation of chronic myeloid leukemia stem cells from normal hematopoietic stem cells at single-cell resolution

**DOI:** 10.1038/s41375-023-01929-6

**Published:** 2023-05-26

**Authors:** Yulin Chen, Susanne Möbius, Konstantin Riege, Steve Hoffmann, Andreas Hochhaus, Thomas Ernst, Karl Lenhard Rudolph

**Affiliations:** 1 Research Group on Stem Cell Aging, Leibniz Institute on Aging–Fritz Lipmann Institute (FLI), 07745 Jena, Germany; 2 Abteilung Hämatologie und Internistische Onkologie, Klinik für Innere Medizin II, Universitätsklinikum Jena, 07747 Jena, Germany; 3 The Computational Biology Group, Leibniz Institute on Aging–Fritz Lipmann Institute (FLI), 07745 Jena, Germany; 4 Faculty of Medicine, University Hospital Jena (UKJ), Friedrich Schiller University, Jena, Germany

**Keywords:** Chronic myeloid leukaemia, Haematopoietic stem cells

## To the Editor:

Chronic myeloid leukemia (CML) originates from hematopoietic stem cells (HSC), which is causally linked to a reciprocal translocation on chromosomes 9 and 22, referred to as the Philadelphia chromosome (Ph), resulting in a *BCR::ABL1* gene fusion and aberrant activation of tyrosine kinase signaling [1]. Treatment with tyrosine kinase inhibitors (TKI) effectively removes the majority of leukemic cells and drives the disease into deep molecular remission, but residual CML cells remain and relapses occur in a significant percentage of patients after discontinuation of TKI treatment, suggesting survival of CML stem cells (CML-SC) at remission stage [2, 3]. It has been shown that CML-SC in chronic phase express the same surface markers (Lin^−^CD34^+^CD38^−^) as normal HSC [4]. In order to distinguish CML-SC from normal HSC, several cell surface markers were identified based on *BCR::ABL1* enrichment, including CD25, CD26, CD33, CD93, and IL1RAP [5–9]. However, it would be important to conduct single-cell genetic analysis to determine the correlation between CML-SC marker-positivity and the presence of the *BCR::ABL1* gene fusion at DNA level as well as its correlation with additional, genetic mutations that co-segregate with CML-SC *versus* normal HSC.

**Fig. 1:**
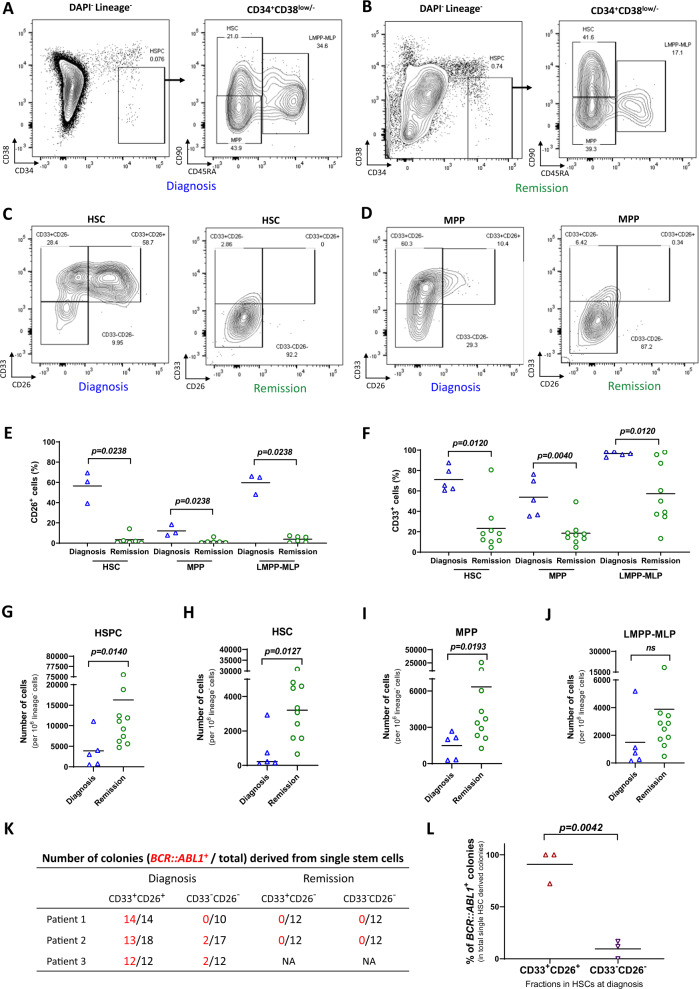
Co-expression of CD33 and CD26 identified *BCR::ABL1*^*+*^ CML-SC at chronic phase. **A**–**J** Analysis on the immunophenotypic subpopulations in hematopoietic stem and progenitor cells (HSPC) from CML patients at diagnosis vs. remission stage (3 or 6 months after initiation of TKI treatment) by FACS. **A**, **B** Representative FACS profiles and gating strategies of HSPC (Lin^−^CD34^+^CD38^low/−^), HSC (Lin^−^CD34^+^CD38^low/−^CD45RA^−^CD90^+^), MPP (Lin^−^CD34^+^CD38^low/−^CD45RA^−^CD90^−^), and LMPP-MLP (Lin^−^CD34^+^CD38^low/−^CD45RA^+^CD90^low/−^), at **A** diagnosis vs. **B** remission stage. Representative FACS profiles of (**C**) HSC and (**D**) MPP at diagnosis vs. remission stage with additional markers of CD33 and CD26. **E** Quantification of the percentage of CD26^+^ fractions in HSC, MPP, and LMPP-MLP at diagnosis (*n* = 3) vs. remission (*n* = 6) stage. **F** Quantification of the percentage of CD33^+^ fraction in HSC, MPP, and LMPP-MLP at diagnosis (*n* = 5) vs. remission (*n* = 9) stage. **E**, **F** Data are not normally distributed (Shapiro–Wilk test); *p* value was calculated by Mann–Whitney U test. Quantification of the number of cells per 10^6^ cells in **G** HSPC, **H** HSC, **I** MPP and **J** LMPP-MLP fractions from CML patients at diagnosis (*n* = 5) vs. remission (*n* = 10). Data are not normally distributed (Shapiro–Wilk test); *p*-values were calculated by Mann–Whitney U test. **K**, **L** FACS-purified single CML-SC (CD33^+^CD26^+^Lin^−^CD34^+^CD38^low/−^CD45RA^−^CD90^+^) or normal HSC (CD33^−^CD26^−^Lin^−^CD34^+^CD38^low/−^CD45RA^−^CD90^+^) from BM aspirates of CML patients at diagnosis and single CD33^+^CD26^−^ and CD33^−^CD26^−^ HSC from BM aspirates of CML patients at remission were seeded into 96-wells plates and cultured for 3 weeks. *BCR::ABL1* genotyping was performed on DNA from single-cell-derived colonies by specifically designed primers for individual patients. **K** The number of *BCR::ABL1*^+^ colonies (in red) and total investigated colonies (in black) are shown. **L** Percentage of *BCR::ABL1*^+^ colonies out of total number of genotyped colonies for each population are depicted for CD33^+^CD26^+^ CML-SC and CD33^−^CD26^−^ HSC at diagnosis (*n* = 3). Data are normally distributed (Shapiro–Wilk test); *p*-values were calculated by unpaired t-test with Welch’s correction. **E**–**J** and **L** Data are depicted as mean and individual values, ns not significant.

For this purpose, we firstly analyzed the immunophenotypes of hematopoietic stem and progenitor cells (HSPC) in bone marrow aspirates from CML patients at diagnosis and hematologic remission, by staining for cell surface antigens of lineage markers, CD34, CD38, CD90, and CD45RA, together with CD26 and CD33. CD26 is a promising Ph^+^ CML-SC marker that was recently identified, highly expressed at newly diagnosed CML patients but declined after TKI treatment [6, 10]. CD33 is an antigen, expressed on myeloid cells and myeloid progenitor cells, as well as on HSC-enriched cell populations [11]. In a recent study, CD33 was found also highly expressed on CML-SC [7]. As described [6, 7, 10], we found the immunophenotypic HSC (Lin^−^CD34^+^CD38^low/−^CD90^+^CD45RA^−^) in CML patients contain a high percentage of CD26^+^/CD33^+^ cells at diagnosis, which strongly declined at remission stage after 3 or 6 months TKI treatment (Fig. 1A–F). Similar changes were also observed in multipotent progenitor cells (MPP) and lymphoid-primed multipotent progenitor containing multi-lymphoid progenitors (LMPP-MLP, Fig. 1A–F). The percentage of CD33^+^ cells in LMPP-MLP population was higher than that in HSC or MPP populations at diagnosis. CD33-expression also remained elevated in LMPP-MLP after TKI treatment despite a significant therapy-induced reduction (Fig. 1F). The total number of HSPC increased in bone marrow of the patients at remission compared to diagnosis stage (Fig. 1G–J), indicating that CML-SC suppress normal hematopoiesis, which is alleviated by TKI-therapy.

To enable single-cell analysis, single CML-SC (Lin^−^CD34^+^CD38^low/−^CD90^+^CD45RA^−^CD33^+^CD26^+^) and normal HSC (Lin^−^CD34^+^CD38^low/−^CD90^+^CD45RA^−^CD26^−^) purified by Fluorescence Activated Cell Sorting (FACS) were grown into colonies. To analyze differences in the colony forming efficiency (the number of colonies grown out *per* number of single-cell seeded wells), following three subtypes of HSC were investigated: (i) CD33^+^CD26^+^, (ii) CD33^+^CD26^−^, and (iii) CD33^−^CD26^−^ HSC. Analysis at diagnosis revealed that CD33^+^CD26^+^ HSC had the highest colony forming efficiency (33.3%), followed by CD33^−^CD26^−^ HSC (24.2%) and CD33^+^CD26^−^ HSC (3.3%). At remission stage, the colony forming efficiency of CD33^+^CD26^−^ HSC was 25.4%, and 28.3% for CD33^−^CD26^−^ HSC, whereas CD33^+^CD26^+^ HSC were not detectable and the colony forming capacity could not be assessed.

Patient-specific genomic polymerase chain reaction (PCR) showed that CD33^+^CD26^+^ HSC derived colonies were enriched for *BCR::ABL1-*positive status (Fig. 1K, L). In contrast, all colonies derived from CD33^−^CD26^−^ HSC or CD33^+^CD26^−^ HSC analyzed at remission stage were *BCR::ABL1-*negative (Fig. 1K). Together, these results provide genetic evidence at single-cell level, supporting the concept that CD26/CD33 co-expression separates CML-SC from normal HSC in CML patients at chronic phase. The genetic analysis of CD33^−^CD26^−^ HSC and CD33^+^CD26^−^ HSC at remission stage indicates that both populations represent non-leukemic, *BCR::ABL1*-negative HSC. CD33-positivity has been associated with human HSC and myeloid progenitors [11]. Thus, it is possible that CD33-positivity at this stage marks a myeloid-primed subpopulation of non-leukemic HSC. The data on colony formation suggest that the regeneration capacity of CD33^+^CD26^−^ HSC is strongly repressed by CML-SC at diagnosis stage.

**Fig. 2:**
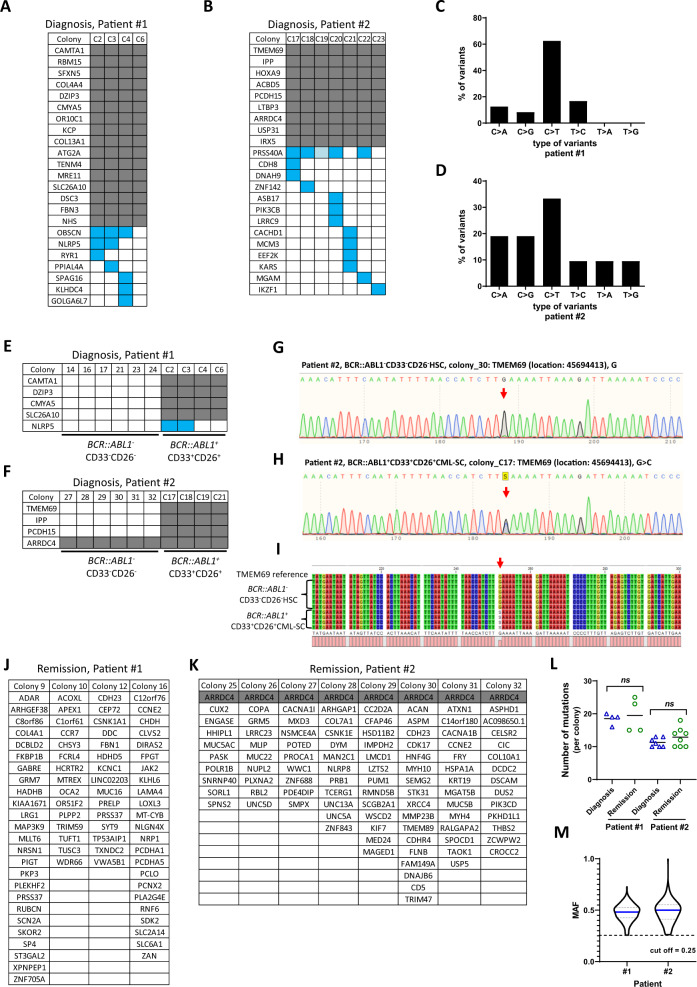
Detection of mutations in *BCR::ABL1* positive CML-SC and *BCR::ABL1* negative HSC from CML patients before and after TKI treatment. **A**–**D** FACS-purified single CML-SC (CD33^+^CD26^+^Lin^−^CD34^+^CD38^low/−^CD45RA^−^CD90^+^) from CML patients (Patient #1 & #2) at diagnosis were seeded into 96-wells plates and cultured for 3 weeks. *BCR::ABL1*^+^ colonies were confirmed by genomic PCR and used for WES to determine somatic mutations in individual colonies, together with WES of buccal swabs DNA to exclude germline mutations for each patient. The gene symbols containing somatic mutations are listed for the colonies that were investigated of (**A**) patient #1 and (**B**) patient #2. Gray frames indicate the same single nucleotide variants that are present and recurrent in every colony of the individual patient. Blue frames indicate single nucleotide variants that are not recurrent in all the investigated colonies. Light blue one (C19, Patient #2) indicates MAF was lower than 0.25. Percentage of different types of single nucleotide variants in (**C**) patient #1 and (**D**) patient #2 at diagnosis. **E**–**I** Randomly selected mutated genes identified by WES in **A** and **B** were confirmed by Sanger sequencing using DNA of the indicated *BCR::ABL1*^−^ HSC and *BCR::ABL1*^+^ CML-SC derived colonies from (**E**) patient #1 and (**F**) patient #2 at diagnosis. Gray/blue frames indicate mutations that were identified by WES and validated by Sanger sequencing, while the white frames represent the colonies where no mutation was detected by Sanger sequencing. Representative profiles of Sanger sequencing of *TMEM69* in **G** *BCR::ABL1*^−^ HSC colony (wild-type) and **H** *BCR::ABL1*^+^ CML-SC colony (mutated, G > C). **I** Alignment of DNA sequences of *BCR::ABL1*^−^ colonies (#27, 28, 29, 30, 31, 32) and *BCR::ABL1*^+^ colonies (#C17, C18, C19, C21) from patient #2 detected by Sanger sequencing. Red arrow indicates the mutant nucleotide. The list of mutant genes identified by the same method as used in **A** and **B** of individual colonies from (**J**) patient #1 and (**K**) patient #2 at remission after 3-month TKI therapy. Gray frames indicate the same single nucleotide variants that are present and recurrent in every colony of the patient. **L** Number of mutant genes per colony was detected by WES in patient #1 and #2 at diagnosis (CML-SC colonies) and remission (HSC colonies). Data are normally distributed (Shapiro–Wilk test); *p*-values were calculated by unpaired t-test with Welch’s correction. Data are depicted as mean and individual values, ns not significant. **M** MAF was calculated with the reads of mutant alleles divided by the total reads in a specific nucleotide for individual patients. The medians of reads of all the analyzed mutations was 48 for patient #1 and 45 for patient #2. To exclude mutations induced by cell culture, the cut-off of MAF in all the analyzed mutations was set to 0.25. Date was depicted as violin plot with median (in blue) and quartiles.

Previous studies indicate that somatic mutations occur in Ph^+^ and Ph^−^ cells of CML patients [12, 13]. To determine mutations at diagnosis, bone marrow aspirates of two patients were genotyped for 54 commonly mutated leukemia-associated genes by targeted next-generation sequencing (NGS) according to our previous protocols [14]. This analysis did not reveal any mutation in these two patients. To identify somatic mutations at stem cell level, whole exome sequencing (WES) of single CML-SC or single normal HSC derived colonies was conducted (EGA accession ID: EGAS00001006904) using DNA from buccal swabs as germline control. Only mutations in exons of protein coding genes with a coverage of >10 reads and a mutant allele fraction (MAF) of ≥0.25 were used for subsequent analysis. The WES data revealed that different CML-SC colonies at diagnosis carried a unifying, patient-specific set of mutations in each of the two patients (Fig. 2A, B, in gray). The unifying mutations affected all colonies of the individual patients at the exact same gene locus, indicating a patient-specific clonal mutation profile in *BCR::ABL1*^+^ CML-SC. Moreover, additional mutations developed in a subset of colonies of the patients (Fig. 2A, B, in blue). In most of the cases, these additional mutations were only present in one of the CML-SC derived colonies, suggesting that these mutations may not exhibit a clonal advantage. Three mutations occurred in multiple sequenced colonies (Fig. 2A, B*OBSCN, NLRP5*, and *PRSS40A*), suggesting that these mutations may have had a selective advantage within the clonally expanding CML-SC pool. In general and in line with previous reports [15], C to T mutations were most frequent (Fig. 2C, D).

Randomly selected mutations in CML-SC derived colonies were validated by Sanger sequencing (Fig. 2E, F: right side: *BCR::ABL1*^+^CD33^+^CD26^+^). Colonies from non-transformed *BCR::ABL1*^−^ HSC (CD33^−^CD26^−^ HSC) at diagnosis before TKI treatment did not carry CML-SC-specific mutations (Fig. 2E, F: left side: *BCR::ABL1*^−^CD33^−^CD26^−^), except for one mutation in the *ARRDC4* gene in patient #2 (Fig. 2E–I). This *ARRDC4* mutation was present in all analyzed clones, suggesting that it had most likely occurred during early embryonic development of the hematopoietic system. In addition, WES of normal *BCR::ABL1*^−^ HSC (including CD33^+^CD26^−^ and CD33^−^CD26^−^ HSC derived colonies) at remission stage from the same patients after TKI treatment did also not show CML-SC-specific mutations, or any other clonally expanding mutation, but distinct sets of gene mutations randomly occurring in individual HSC-derived colonies (Fig. 2J, K). No significant difference was found in the number of mutations in colonies derived from CD33^+^CD26^−^ HSC *versus* CD33^−^CD26^−^ HSC at remission stage. These data indicate that clonal mutation occurred in *BCR::ABL1*^+^ CML-SC before TKI treatment, but there was no evidence for pre-leukemic clonal mutations in *BCR::ABL1*^−^ HSC of these two patients. The total number of gene mutations showed no significant change in CML-SC *versus* HSC derived colonies (Fig. 2L). The medians of MAF in WES analysis of all analyzed colonies were 0.48 (Patient #1) and 0.50 (Patient #2), suggesting that the mutations were present in the originally single-cell sorted stem cells (Fig. 2M).

In summary, this study supports the concept that co-expression of CD26/CD33 markers effectively discriminates *BCR::ABL1*^*+*^ CML-SC from normal *BCR::ABL1*^*−*^ HSC in chronic phase CML. Single-cell analysis demonstrates that CML-SC carry a unifying set of patient specific, recurrent, clonal mutations. In contrast, *BCR::ABL1*^−^ HSC from the same patients show no evidence for recurrent, clonal mutations, aside of 1 mutation in patient #2 that was present in all clones likely stemming from an early stage of blood development (Fig. 2F). These data indicate the pre-leukemic HSC of CML origin, which acquired the disease-inducing *BCR::ABL1* fusion gene, harbored a unique set of pre-existing non-clonal gene mutations to start with. Alternatively, it is also possible that some of the underlying recurrent mutations in the CML-SC pool were actually acquired after the *BCR::ABL1* formation and these mutations had positive effects in disease evolution and eventually took over the CML-SC pool. Based on the high-quality reads, the data of this study found evidence for such a clonal evolution, showing the same mutations only across a fraction of colonies derived from individual CML-SC (Fig. 2A, B*OBSCN, NLRP5, PRSS40A*). These findings indicate that selection processes of newly acquired mutations can occur within the pool of CML-SC.

Our analysis of normal HSC from the two CML patients of this study showed no evidence of a pre-leukemic HSC subpopulation (*BCR::ABL1*^*−*^ HSC) that harbored clonal mutations at either diagnosis or remission. Also an analysis of aging/leukemia-associated mutations in hotspot genes (e.g. *DNMT3A, TET2, ASXL1, RUNX1, TP53 etc*.) [12–15] did not reveal an underlying mutation in any of these genes in the normal HSC or CML-SC population of the two patients. While the average occurrence of such mutations in CML patient is 20–30% at diagnosis [12, 13], it can be assumed that the absence of such mutations in our study was just by chance.

In conclusion, this study provides experimental evidence that CD26/CD33 co-expression discriminates genetically defined CML-SC from non-leukemic HSC in CML patients. The here described approach enables researchers to determine the clonal evolution of CML-SC at single-cell dimension during chronic disease stage, progression, and relapse.

## Supplementary information

Supplementary methods

## Data Availability

The whole exome DNA sequencing data have been deposited in the European Genome-phenome Archive (EGA) with accession ID: EGAS00001006904.
