# Supplementary material for: Genetic separation of chronic myeloid leukemia stem cells from normal hematopoietic stem cells at single-cell resolution
Source: Leukemia. 2023 May 26;37(7):1561–6. doi: 10.1038/s41375-023-01929-6 (PMC10317832; doi:10.1038/s41375-023-01929-6)
Supplement: Supplementary file 1 — Supplementary methods [file 41375_2023_1929_MOESM1_ESM.docx]

**Genetic separation of chronic myeloid leukemia stem cells from normal hematopoietic stem cells at single-cell resolution**

Yulin Chen^1^, Susanne Möbius^2^, Konstantin Riege^3^, Steve Hoffmann^3^, Andreas Hochhaus^2^, Thomas Ernst^2,5^, K. Lenhard Rudolph^1,4,5^

1. Research Group on Stem Cell Aging, Leibniz Institute on Aging – Fritz Lipmann Institute (FLI), Jena, Germany

2. Abteilung Hämatologie und Internistische Onkologie, Klinik für Innere Medizin II, Universitätsklinikum Jena, Jena, Germany

3. The Computational Biology Group, Leibniz Institute on Aging – Fritz Lipmann Institute (FLI), Jena, Germany

4. Faculty of Medicine, University Hospital Jena (UKJ), Friedrich Schiller University, Jena, Germany

5. These authors contributed equally

Keywords: Chronic myeloid leukemia, leukemia stem cells, clonal mutation, *BCR::ABL1* genotyping

Correspondence: Thomas Ernst, E-mail: thomas.ernst@med.uni-jena.de; K. Lenhard Rudolph, E-mail: [Lenhard.Rudolph@Leibniz-Fli.de](mailto:Lenhard.Rudolph@Leibniz-Fli.de)

**Methods**

**Patients**

Fifteen bone marrow (BM) aspirates were obtained at diagnosis (n=5) or remission stage (n=10) from 10 CML patients (median age 51 years, range: 20-77 years). For 4 patients, paired samples (diagnosis versus remission) were analyzed on the expression of immunophenotypic markers. For 2 patients, paired samples (diagnosis versus remission) were used for whole exome sequencing (WES). Remission samples were investigated 3 or 6 months after treatment with the TKI nilotinib or dasatinib (n=5, each). The study has been approved by the institutional ethics committee of Jena University Hospital and patients were investigated after written informed consent in accordance with the Declaration of Helsinki.

***BCR::ABL1* genotyping**

Genomic DNA was extracted from peripheral blood samples taken at the time point of initial diagnosis. To identify the patient-specific BCR::ABL1 breakpoint, Illumina’s Nextera Rapid Capture Custom Enrichment assay covering both involved genes completely was used (covered region ABL1: chr9:133,589,068-133,763,247; NCBI17/hg19; covered region BCR: chr22:23,522,352-23,660,391; NCBI17/hg19). Sequencing data were analyzed using NextGENe Software (Softgenetics). Primers enclosing the junction of both genes were designed for each patient and breakpoint regions were amplified by PCR from DNA isolated from individual single CML-SC and HSC derived colonies.

**NGS analysis of paired samples**

DNA sequencing of exome samples from single-cell derived clones and patient buccal swabs as control to distinguish germline and somatic mutations was performed using Illumina’s next-generation sequencing (NGS) methodology.^1^ Briefly, total DNA was quality checked by Agilent 2100 Bioanalyzer Instrument (High Sensitivity DNA kit) and quantified by Quant-iT™ PicoGreen™. Libraries were prepared from 200 ng of input material using SureSelect Human All Exon V6 (manufacturer's instructions) and subsequently quantified and quality checked by Agilent 2100 Bioanalyzer Instrument (DNA 7500 kit). Libraries were pooled and sequenced on NextSeq 500 System (High Output Flow Cell) running in 150 cycles (2x 75bp paired-end) mode. Sequence information was converted to FASTQ format using bcl2fastq v2.20.0.422.

**Flow cytometry**

Mononuclear cells were isolated from bone marrow aspirates using Lymphoprep (Stem cell technologies, Vancouver, BC, Canada), and lineage negative cells were enriched using Human Hematopoietic Lineage Depletion Kit (Thermo Fisher Scientific, Waltham, MA USA), anti-biotin microbeads and MACS Separation LS Columns (Miltenyi Biotec, Bergisch Gladbach, Germany). For sorting hematopoietic stem and progenitor cells (HSPCs), previously published marker combination were used^2, 3^: lineage-negative cells were stained with CD90-FITC, CD26-PE, CD38-PerCP/Cy5.5, CD45RA-PE/Cy7, CD34-APC, Streptavidin-APC/Cy7, CD33-BV605 antibodies (Biolegend, San Diego, California, USA). HSPCs were sorted by flow cytometer Aria III (BD Biosciences, Franklin Lakes, New Jersey, USA).

**Colony forming assay and DNA extraction**

Single CML-SC or HSC were sorted into 96 well U-bottom plates with 150ul SFEM medium (Stem cell technologies) supplemented with recombinant human TPO (50ng/ml), SCF (50ng/ml), IL-3 (20ng/ml), IL-6 (20ng/ml), GCSF (20ng/ml) (Thermo Fisher Scientific), penicillin (50U/ml)/streptomycin (50ug/ml), 10% FBS, and cultured at 37 °C with 5% CO_2_. Colonies were counted and harvested 3 weeks after seeding. Cell pallet was lysed at 55 °C for 4 h, using 200ul lysis buffer containing 10mM Tris-HCl, 2mM EDTA, 0.5% SDS, 100mM NaCl, and proteinase K. The cell lysate was further incubated with RNase A at 37 °C for 30 min. DNA was extracted using Phenol: Chloroform: Isoamyl Alcohol 25:24:1 (Merck, Darmstadt, Germany), followed by ethanol precipitation.

**Mutation calling**

Variant calling was conducted by the in-house pipeline Muvac [https://github.com/Hoffmann-Lab/muvac] v0.7.1 which follows the GATK^4, 5^ somatic mutation calling best practices. Trimmomatic^6^ v0.38 (5nt sliding window approach, mean quality cutoff 22) was utilized for read quality trimming according to inspections made from FastQC [https://www.bioinformatics.babraham.ac.uk/projects/fastqc/] v0.11.8. Subsequently, processed sequencing data was aligned to the human reference genome hg38, retrieved along with its gene annotation from Ensembl v92. Alignments were performed with segemehl^7, 8^ v0.3.4 (95% accuracy, read splitting disabled) and filtered by SAMtools^9^ v1.10 for uniqueness and properly aligned mate pairs. After removal of duplicated reads with Picard MarkDuplicates [https://broadinstitute.github.io/picard] v2.23.4, overlapping mate pair sequences were cut from the second mate utilizing BamUtil clipOverlap^10^ v1.0.14. Further, indels were left aligned and base quality scores were re-calibrated utilizing GATK v4.1.4.1 LeftAlignIndels and BaseRecalibrator/ApplyBQSR trained on known sites from NCBI retrieved dbSNP v151. Given gnomAD^11^ population derived genomic variants from GATK Resource Bundle “gatk-best-practices” and a manually created panel of normals from common variants called in Mutect2 normal-only mode, systematic errors within the sequencing data was addressed while running Mutect2 on matched tumor-normal-pairs (minimum coverage 10, disabled Multi-Nucleotide Polymorphisms, collecting read orientation metrics). The received raw sets of somatic variants were filtered and normalized in downstream processes. To account for strand biases and possible cross-sample contamination, FilterMutectCalls was supplied with the resulting table of GATK LearnReadOrientationModel, given the prior calculated read orientation metrics, and the predicted contamination from GetPileupSummaries on ExAC^12^ common homozygous germline haplotypes, shipped as part of GATK Resource Bundle “gatk-best-practices”. Multiallelic sites were split by BCFtools norm^13^ v1.10, allele frequencies and allele counts corrected accordingly utilizing vcflib vcffixup^14^ v1.0.1. Finally, indels were trimmed and left-aligned using Vt normalize^15^ v0.57721.

**Mutation validation**

Primers were designed to specifically amplify 200-400bp DNA within the gene regions where the mutations are located. PCR reaction was conducted using Phusion™ High-Fidelity DNA Polymerase (Thermo Fisher Scientific) and the original DNA from cell colonies as templates. Amplicons were purified by Wizard® SV Gel and PCR Clean-Up System (Promega, Madison, Wisconsin, USA). Sanger sequencing was conducted by Microsynth Seqlab GmbH, Göttingen, Germany. The primers for amplicons were also used for Sanger Sequencing. Sequencing results was analyzed using SnapGene Viewer and sequence alignment was performed using CLC Sequence Viewer.

**Statistical analysis**

Normal distribution of all the data was tested by Shapiro-Wilk test. Statistical testing of normally distributed data was performed using unpaired t-test with Welch’s correction. Non-normally distributed data was assessed using the Mann-Whitney U test.

**References**

1. Bentley DR, Balasubramanian S, Swerdlow HP, Smith GP, Milton J, Brown CG*, et al.* Accurate whole human genome sequencing using reversible terminator chemistry. *Nature* 2008 Nov 6; **456**(7218)**:** 53-59.

2. Karamitros D, Stoilova B, Aboukhalil Z, Hamey F, Reinisch A, Samitsch M*, et al.* Single-cell analysis reveals the continuum of human lympho-myeloid progenitor cells. *Nature immunology* 2018 Jan; **19**(1)**:** 85-97.

3. Herrmann H, Sadovnik I, Cerny-Reiterer S, Rülicke T, Stefanzl G, Willmann M*, et al.* Dipeptidylpeptidase IV (CD26) defines leukemic stem cells (LSC) in chronic myeloid leukemia. *Blood* 2014 Jun 19; **123**(25)**:** 3951-3962.

4. McKenna A, Hanna M, Banks E, Sivachenko A, Cibulskis K, Kernytsky A*, et al.* The Genome Analysis Toolkit: a MapReduce framework for analyzing next-generation DNA sequencing data. *Genome research* 2010 Sep; **20**(9)**:** 1297-1303.

5. DePristo MA, Banks E, Poplin R, Garimella KV, Maguire JR, Hartl C*, et al.* A framework for variation discovery and genotyping using next-generation DNA sequencing data. *Nature genetics* 2011 May; **43**(5)**:** 491-498.

6. Bolger AM, Lohse M, Usadel B. Trimmomatic: a flexible trimmer for Illumina sequence data. *Bioinformatics (Oxford, England)* 2014 Aug 1; **30**(15)**:** 2114-2120.

7. Hoffmann S, Otto C, Doose G, Tanzer A, Langenberger D, Christ S*, et al.* A multi-split mapping algorithm for circular RNA, splicing, trans-splicing and fusion detection. *Genome biology* 2014 Feb 10; **15**(2)**:** R34.

8. Hoffmann S, Otto C, Kurtz S, Sharma CM, Khaitovich P, Vogel J*, et al.* Fast mapping of short sequences with mismatches, insertions and deletions using index structures. *PLoS computational biology* 2009 Sep; **5**(9)**:** e1000502.

9. Li H, Handsaker B, Wysoker A, Fennell T, Ruan J, Homer N*, et al.* The Sequence Alignment/Map format and SAMtools. *Bioinformatics (Oxford, England)* 2009 Aug 15; **25**(16)**:** 2078-2079.

10. Jun G, Wing MK, Abecasis GR, Kang HM. An efficient and scalable analysis framework for variant extraction and refinement from population-scale DNA sequence data. *Genome research* 2015 Jun; **25**(6)**:** 918-925.

11. Karczewski KJ, Francioli LC, Tiao G, Cummings BB, Alföldi J, Wang Q*, et al.* The mutational constraint spectrum quantified from variation in 141,456 humans. *Nature* 2020 May; **581**(7809)**:** 434-443.

12. Karczewski KJ, Weisburd B, Thomas B, Solomonson M, Ruderfer DM, Kavanagh D*, et al.* The ExAC browser: displaying reference data information from over 60 000 exomes. *Nucleic acids research* 2017 Jan 4; **45**(D1)**:** D840-d845.

13. Li H. A statistical framework for SNP calling, mutation discovery, association mapping and population genetical parameter estimation from sequencing data. *Bioinformatics (Oxford, England)* 2011 Nov 1; **27**(21)**:** 2987-2993.

14. Garrison E, Kronenberg ZN, Dawson ET, Pedersen BS, Prins P. Vcflib and tools for processing the VCF variant call format. *bioRxiv* 2021**:** 2021.2005.2021.445151.

15. Tan A, Abecasis GR, Kang HM. Unified representation of genetic variants. *Bioinformatics (Oxford, England)* 2015; **31**(13)**:** 2202-2204.
